# Genetic risk assessment of thrombophilia in patients with adverse obstetric outcomes

**DOI:** 10.1371/journal.pone.0211114

**Published:** 2019-02-27

**Authors:** M. Fernández Arias, E. Mazarico, A. Gonzalez, M. Muniesa, C. Molinet, L. Almeida, M. D. Gómez Roig

**Affiliations:** 1 BCNatal—Barcelona Center for Maternal-Fetal and Neonatal Medicine (Hospital Sant Joan de Deu and Hospital Clínic), Barcelona, Spain; 2 Maternal and Child Health and Development Network II (SAMID II) funded by Instituto de Salud Carlos III (ISCIII)—Sub-Directorate General for Research Assessment and Promotion and the European Regional Development Fund (ERDF), Madrid, Spain; 3 Institut de Recerca Sant Joan de Déu (IR-SJD), Barcelona, Spain; Royal College of Surgeons in Ireland, IRELAND

## Abstract

**Objectives:**

To investigate the incidence of inherited thrombophilias in patients with adverse obstetric outcomes and to compare detection rates of thrombophilias between standard blood tests and a novel genetic test.

**Methods:**

This is a case-control prospective study performed in Hospital Sant Joan de Déu in Barcelona, Spain. Cases had a history of intrauterine growth restriction requiring delivery before 34 weeks gestation, placental abruption before 34 weeks gestation, or severe preeclampsia. Controls had at least two normal, spontaneously conceived pregnancies at term, without complications or no underlying medical disease. At least 3 months after delivery, all case and control women underwent blood collection for standard blood tests for thrombophilias and saliva collection for the genetic test, which enables the diagnosis of 12 hereditary thrombophilias by analyzing genetic variants affecting different points of the blood coagulation cascade.

**Results:**

The study included 33 cases and 41 controls. There were no statistically significant differences between cases and controls in the standard blood tests for thrombophilias in plasma or the TiC test for genetic variables. One clinical-genetic model was generated using variables with the lowest P values: ABO, body mass index, C_rs5985, C_rs6025, and protein S. This model exhibited good prediction capacity, with an area under the curve of almost 0.7 (P <0.05), sensitivity of almost 67%, and specificity of 70%.

**Conclusion:**

Although some association may exist between hypercoagulability and pregnancy outcomes, no significant direct correlation was observed between adverse obstetric outcomes and inherited thrombophilias when analyzed using either standard blood tests or the genetic test. Future studies with a larger sample size are required to create a clinical-genetic model that better discriminates women with a history of adverse pregnancy outcomes and an increased risk of poor outcomes in subsequent pregnancies.

## Introduction

Thrombophilia describes a tendency to develop thromboses because of inherited or acquired disorders of blood coagulation or fibrinolysis, which lead to a prothrombotic state [1, 2]. Causes of hereditary thrombophilia include factor V Leiden mutation (activated protein C [PC] resistance); prothrombin 20210A mutation; PC, protein S (PS), and antithrombin III (AT III) deficiencies; lupus anticoagulant; anticardiolipin antibodies; methylenetetrahydrofolate (*MTHFR*) gene mutation; and hyperhomocysteinemia[1].

The hemostatic system appears to play an important role in both establishing and maintaining pregnancy. Development of the placental circulation is ensured by structural modifications of the spiral arteries and a pregnancy-induced hypercoagulable state resulting from an increase in procoagulant factors and a decrease in anticoagulant factors and fibrinolysis [2,3, 4]. Thus, pregnancy itself induces a physiological hypercoagulable state that might be exacerbated by inherited or acquired thrombophilia [5].

There has always existed much controversy about the association between inherited thrombophilias and the possibility of adverse pregnancy outcomes due to uteroplacental thrombosis [6, 7]. A number of authors suggest that the presence of any thrombophilic condition may cause venous or arterial thrombosis and placental circulation abnormalities, leading to a higher rate of certain obstetrical complications, such as fetal loss, severe preeclampsia, severe fetal growth restriction, or placental abruption [2, 5, 8]. Padmashree et al.[9] reported that inherited and acquired thrombophilias can be found in 49% to 65% of women with pregnancy complications, in contrast to 28% to 22% of women with normal pregnancies, suggesting a three- to eight-fold increased risk of thrombophilia in women with complications. Accordingly, these authors recommended antenatal screening for congenital and acquired thrombophilias among women with prior adverse pregnancy outcomes, with the intention of offering treatment with anticoagulants or antiplatelet agents. Most studies supporting this approach hypothesize that microthrombi, thrombosis, and infarction of the placenta contribute to pregnancy complications or loss [10,11]. In addition, there is evidence that women with any type of thromboembolic defect have a higher prevalence of pregnancy complications [12,13].

Nevertheless, many current guidelines do not recommend screening unless a personal or strong family history of venous thromboembolism is present. Some reasons can be found against screening women with a history of adverse pregnancy outcome. First of all, some studies fail to demonstrate a strong association between hypercoagulability and pregnancy outcomes. And moreover, most pregnant women with inherited thrombophilia have normal pregnancy outcomes [14].

Anticoagulants and aspirin are the best studied and most commonly used therapeutic agents to prevent pregnancy complications in thrombophilic women [2, 15], and they have been demonstrated to reduce the incidence of adverse obstetrical events in women with previous adverse pregnancy outcomes and inherited or acquired thrombophilias [2, 15]. Low molecular weight heparin is often the main treatment for women with thrombophilia and pregnancy complications to prevent adverse pregnancy outcomes [15–19].

In addition to conventional thrombophilias (Factor V Leiden mutation; prothrombin 20210A mutation; PC, PS, and AT III deficiencies; lupus anticoagulant; and anticardiolipin antibodies), novel thrombophilias have been recently investigated and also appear to potentially affect pregnancy outcomes [15]. It is a new technique that analyzes genetic variants affecting different points of the blood coagulation cascade, enabling the diagnosis of 12 hereditary thrombophilias. These variants involve Factor V Leiden (Arg506Gln FV conventional, Arg306Thr FV Cambridge, Arg306Gly FV Hong Kong), Factor II (G20210A), Factor XII (46C>T), Group ABO (rs8176719, rs7853989, rs8176743, rs8176750), Serpina A10 (Arg67Stop), Serpina C1 (Cambridge Ala384Ser II), and Factor XIII (Val34Leu).

The aim of this study was to investigate the incidence of inherited thrombophilias in patients with adverse obstetric outcomes (severe fetal growth restriction, placental abruption, or severe preeclampsia) and to compare detection rates of thrombophilia between standard blood tests and the genetic test, with the goal of improving prediction of patients who could benefit from anticoagulant therapy in subsequent pregnancies.

## Materials and methods

This is a case-control prospective study performed in Hospital Sant Joan de Deú in Barcelona, Spain. The study protocol was approved by the Institutional Review Board of Sant Joan de Déu University Hospital. Written informed consent was obtained from each participant.

### Subjects

Cases and controls were recruited over a 12-month period from women above age 18 (22–45 years old) attending the outpatient clinic in the obstetrics department of the Hospital Sant Joan de Déu and who also gave birth in the same hospital. Gestational ages for all cases and controls were determined by measuring the crown-rump length during the first trimester [20].

Women were eligible for inclusion as cases in this study if they met the following criteria:

1. Intrauterine growth restriction: birth weight <10^th^ percentile with an abnormal uterine arterial Doppler study (pulsatility index >95^th^ percentile [21]) or cerebroplacental ratio (pulsatility index <5^th^ percentile [22]), which led to the need for delivery before 34 weeks gestation.
2. Placental abruption before 34 weeks gestation.
3. Severe preeclampsia [23]: systolic blood pressure (BP) ≥140 mm Hg and/or diastolic BP ≥90 mm Hg measured twice in 6 h after 10 min of rest (determined in the sitting position with the arm at heart level), >300 mg protein in a 24-hour urine collection, and one or more of the following:
  - Systolic BP ≥160 mm Hg and/or diastolic BP ≥110 mm Hg measured on two occasions separated by 6 h, with the patient at rest. Systolic BP >180 and/or diastolic BP >120 mm Hg on two separate occasions in 30 min.
  - Persistent symptoms of eclampsia, including one or more of the following: hyperreflexia with clonus, severe headache, visual disturbances, stupor, epigastric or right upper quadrant pain, nausea, or vomiting.
  - Oliguria: ≤500 mL in 24 h or <100 mL in 3 h, or evidence of renal insufficiency (serum creatinine >1.2 mg/dL or blood urea nitrogen >40 mg/dL).
  - Acute pulmonary edema
  - Hepatic dysfunction
  - Thrombocytopenia (<100,000/mm^3^)
  - Laboratory signs of hemolysis

Women were eligible for inclusion as controls if they met the following criteria: at least two normal, spontaneously-conceived pregnancies; delivery at term without any complications; and no underlying medical disease. Patients with a history of one or more abortions were excluded.

Women were excluded from this study if they exhibited any of the following: illicit drug use, endocrine pathologies that might interfere with fetal growth, previous diagnosis of thrombophilia, treatment with aspirin and/or heparin during their pregnancy, and failure to follow clinical protocols. Other exclusion criteria were multiple-fetus pregnancies, fetal infections, and fetal malformations or genetic anomalies.

### Measurements

Standard blood tests for thrombophilias were performed in all cases and controls at least 3 months after pregnancy. These included tests for Factor V Leiden mutation; prothrombin 20210A mutation; PC and PS deficiencies; lupus anticoagulant; and anticardiolipin antibodies.

At the same time blood was obtained for the standard blood tests, a sample of saliva was obtained from all cases and controls for the genetic test. The Thrombo inCode test was used, as it enables the diagnosis of 12 hereditary thrombophilias (F5 rs6025/rs118203906/rs118203905, F2 rs1799963, F12 rs1801020, F13 rs5985, SERPINC1rs121909548, and SERPINA10 rs2232698 plus the A1 blood group (rs8176719, rs7853989, rs8176743, rs8176750) by analyzing genetic variants affecting different points of the blood coagulation cascade.

### Statistical analysis

A bivariate table was constructed to show the distribution of population characteristics between cases and controls. Normally distributed variables are described as mean and standard deviation and were analyzed using the Student’s t-test. Continuous non-normally distributed variables are presented as median and interquartile range and were analyzed using the Kruskal-Wallis test. Categorical variables are shown as absolute frequency and percentage and were analyzed using the chi-squared test (or Fisher’s exact test when the expected cell frequency was <5). Multivariate logistic regression analysis was performed to explore the relationships between potential risk factors and adverse obstetric events.

The area under the receiver operating characteristic curve (AUC) was used to evaluate the predictive performance of multivariate logistic models. Sensitivity, specificity, and positive and negative likelihood ratios were based on the point of the curve that maximized the Youden index. All calculations were performed using R statistical software (version 3.1.3) (R Development Core Team, 2015). P values <0.05 were considered statistically significant.

## Results

A total of 33 patients were identified as cases and 41 as controls. Table 1 shows the baseline characteristics of each group. There were no significant differences between groups. Cases had a higher body mass index (BMI), but the difference between groups did not reach statistical significance (p = 0.064).

**Table 1:** Baseline characteristics of cases and controls.

	ALL PATIENTS (N = 77)	CONTROLS (N = 44)	CASES (N = 33)	P VALUE	N
History of diabetes	0	0	0	-	77
Smoking				1	77
(no)	72 (93.5%)	41 (93.2%)	31 (93.9%)		
(yes)	5 (6.49%)	3 (6.82%)	2 (6.06%)		
Body Mass Index (BMI)	24.2 (22.0;28.0)	23 (22.0;27.0)	26 (22.0;28.0)	0.064	77
Family background				1	77
(no)	73 (94.8%)	42 (95.5%)	31 (93.9%)		
(yes)	4 (5.19%)	2 (4,55%)	2 (6.06%)		

The incidence of thrombophilias in women with adverse obstetric outcomes (cases group) was 15.2% versus 18.2% in controls. There were no significant differences between both groups.

No significant differences in the results of any standard blood test for thrombophilia were observed between cases and controls (Table 2). Lupus anticoagulant and anticardiolipin antibodies were not compared, as these tests were positive in only 2 and 0 patients, respectively. Moreover, no statistically significant differences in genetic variables were found between the cases and controls (Table 3).

**Table 2:** Results of standard blood tests for thrombophilias in cases and controls.

	ALL PATIENTS(N = 77)	CONTROLS (N = 44)	CASES (N = 33)	P VALUE	N
Protein S	86.0 (18.2)	86.8 (18.4)	85 (18.2)	0.666	77
Activated protein C resistance¤	2.80 (2.50;3.20)	2.70 (2.30;3.20)	2.90 (2.60;3.20)	0.295	77
Lupus anticoagulant				0.180	77
(no)	75 (97.4%)	44 (100%)	31 (93.9%)		
(yes)	2 (2.60%)	0 (0.00%)	2 (6.06%)		
IgG anticardiolipin antibodies (no)	77 (100%)	44 (100%)	33 (100%)	-	77
IgM anticardiolipin antibodies (no)	77 (100%)	44 (100%)	33 (100%)	-	77

**Table 3:** Results of the genetic test in cases and controls.

	ALL PATIENTS (N = 77)	CONTROLS (N = 44)	CASES (N = 33)	P VALUE	N
ABO				0.815	77
0	43 (55.8%)	26 (59.1%)	17 (51.5%)		
1	29 (37.7%)	15 (34.1%)	14 (42.4%)		
2	5 (6.49%)	3 (6.82%)	2 (6.06%)		
FVLPTCOUNT				0.230	77
0	71 (92.2%)	39 (88.6%)	32 (97%)		
1	6 (7.79%)	5 (11.4%)	1 (3.03%)		
C_rs1801020				0.297	77
0	46 (59.7%)	26 (59.1%)	20 (60.6%)		
1	29 (37.7%)	18 (40.9%)	11(33.3%)		
2¤	2 (2.60%)	0 (0.00%)	2 (6.06%)		
C_rs2232698				-	77
0	77 (100%)	44(100%)	33(100%)		
C_rs121909548				-	77
0	77 (100%)	44 (100%)	33 (100%)		
C_rs 6025				0.385	77
0	72 (93.5%)	40 (90.9%)	32 (97%)		
1	5 (6.49%)	4 (9.09%)	1 (3.03%)		
C_rs118203906				-	77
0	77 (100%)	44 (100%)	33 (100%)		
C_rs118203905				-	77
0	77 (100%)	44 (100%)	33 (100%)		
C_rs5985				0.214	77
0	44 (57.1%)	22 (50%)	22 (66.7%)		
1	31 (40.3%)	20 (45.5%)	11 (33.3%)		
2¤	2 (2.60%)	2 (4.55%)	0 (0.00%)		
C_rs1799963				1.000	77
0	76 (98.7%)	43 (97.7%)	33 (100%)		
1¤	1 (1.30%)	1 (2.27%)	0 (0.00%)		

We analyzed multivariate prediction models that considered only thrombophilic variables of usual clinical practice or only genetic variables. None of these models were individually predictive of pregnancy complications. We then generated one clinical-genetic model combining variables with the lowest P values (Tables 4 and 5). This model included ABO, BMI, C_rs5985, C_rs6025, and PS. It exhibited good prediction capacity, with an AUC of almost 0.7 (P <0.05), sensitivity of almost 67%, and specificity of 70%.

**Table 4:** Components of a clinical-genetic model for predicting adverse obstetric outcomes.

Variable	Coefficient	P	Odds ratio	95%CI
ABO	0,32803	0,4357	1,3882	0,6085–3,1672
BMI	0,14672	0,0295	1,1600	1,0147–1,3216
C_rs5985	-0,50241	0,2875	0,6051	0,2397–1,5273
C_rs6025	-1,03036	0,3872	0,3569	0,0345–3,6879
Protein S	-0,018309	0,2241	0,9819	0,9633–1,0113
Constant	-2,32358	0,2029		

**Table 5:** Performance characteristics of a clinical-genetic model (ABO, BMI, C_rs5985, C_rs6025, Protein S) for predicting adverse obstetric outcomes.

AUCC	0,696
CI	0,581–0,796
Sensitivity	66,67
Specificity	70,45
Significance level	0,0015
LR+	2,26
LR-	0,47
Percent of cases correctly classified	66,23

## Discussion

Over the past years, there has been much debate regarding the association between inherited thrombophilias and the risk of placenta-related adverse obstetric outcomes. Many case-control studies and systematic reviews [24–27] have suggested an association. Moreover, an increased risk of recurrent severe pregnancy complications has been reported in women with inherited thrombophilias [28–29]. However, our results did not show a high incidence of inherited thrombophilias in patients with adverse obstetric outcomes (severe fetal growth restriction, placental abruption, or severe preeclampsia). Furthermore, we found that analysis of inherited thrombophilias using the genetic test did not increase detection rates compared with the standard blood tests in patients with adverse obstetric outcomes.

Similar to our results, other studies failed to confirm an association between inherited thrombophilias and adverse obstetric outcomes [30–32], and only weak associations have been found between hypercoagulability and pregnancy outcomes. The lack of association is likely attributable to the multifactorial nature of adverse obstetric outcomes, involving the interaction of epidemiological, clinical, and genetic risk factors. Outcomes do not appear to be solely caused by genetic factors; these factors are common in the general population and do not lead to adverse outcomes in the absence of other risk factors.

Because of discrepancies in the literature regarding the importance of thrombophilias in placenta-related adverse obstetric outcomes, an even larger controversy has recently arisen regarding the use of screening for inherited thrombophilias in women with a history of adverse pregnancy outcomes or loss [33]. The ultimate goal is to be able to identify women with adverse obstetric outcomes who may be candidates for prophylactic heparin or antiplatelet agents in subsequent pregnancies. As our results suggest, the best predictive model includes both clinical and genetic variables. When studying the association between genetic variants and a phenotype (or event), it is often convenient to analyze the association by grouping genetic variants, since in many diseases, the genetic risk consists of multiple variants in different genes; analysis of variants individually usually lacks adequate statistical power because their individual contribution to risk is very low, leading to inconsistent results. This was demonstrated in the current study, as we found no significant differences between cases and controls when variants were evaluated individually. But when this genetic information was grouped together and complemented with clinical information, the test had good predictive capacity for adverse obstetric outcomes.

We note limitations to our study. The main limitation was the small sample size, which may have rendered our study underpowered to evaluate differences in detecting inherited thrombophilias between groups. Another limitation is that sticky platelet syndrome was not included in the evaluation of thrombophilias, on one hand because it is an expensive and operator-dependent test and on the other hand because the vast majority of published studies on hereditary thrombophilias do not consider it.

In conclusion, our study shows that, although some association may exist between hypercoagulability and pregnancy outcomes, no significant direct correlation was observed between adverse obstetric outcomes and inherited thrombophilias when analyzed using either standard blood tests or the genetic test. Future studies with a larger sample size are required to create a clinical-genetic model that better discriminates women with a history of adverse pregnancy outcomes and an increased risk of poor outcomes in following pregnancies.
